# Transcriptome profile analysis of young floral buds of fertile and sterile plants from the self-pollinated offspring of the hybrid between novel restorer line NR1 and *Nsa* CMS line in *Brassica napus*

**DOI:** 10.1186/1471-2164-14-26

**Published:** 2013-01-16

**Authors:** Xiaohong Yan, Caihua Dong, Jingyin Yu, Wanghui Liu, Chenghong Jiang, Jia Liu, Qiong Hu, Xiaoping Fang, Wenhui Wei

**Affiliations:** 1 Oil Crops Research Institute of the Chinese Academy of Agricultural Sciences/Key Laboratory of Biology and Genetic Improvement of Oil Crops, Ministry of Agriculture, Wuhan, 430062, China

## Abstract

**Background:**

The fertile and sterile plants were derived from the self-pollinated offspring of the F_1_ hybrid between the novel restorer line NR1 and the *Nsa* CMS line in *Brassica napus*. To elucidate gene expression and regulation caused by the A and C subgenomes of *B*. *napus*, as well as the alien chromosome and cytoplasm from *Sinapis arvensis* during the development of young floral buds, we performed a genome-wide high-throughput transcriptomic sequencing for young floral buds of sterile and fertile plants.

**Results:**

In this study, equal amounts of total RNAs taken from young floral buds of sterile and fertile plants were sequenced using the Illumina/Solexa platform. After filtered out low quality data, a total of 2,760,574 and 2,714,441 clean tags were remained in the two libraries, from which 242,163 (Ste) and 253,507 (Fer) distinct tags were obtained. All distinct sequencing tags were annotated using all possible CATG+17-nt sequences of the genome and transcriptome of *Brassica rapa* and those of *Brassica oleracea* as the reference sequences, respectively. In total, 3231 genes of *B*. *rapa* and 3371 genes of *B*. *oleracea* were detected with significant differential expression levels. GO and pathway-based analyses were performed to determine and further to understand the biological functions of those differentially expressed genes (DEGs). In addition, there were 1089 specially expressed unknown tags in Fer, which were neither mapped to *B*. *oleracea* nor to *B*. *rapa*, and these unique tags were presumed to arise basically from the added alien chromosome of *S*. *arvensis*. Fifteen genes were randomly selected and their expression levels were confirmed by quantitative RT-PCR, and fourteen of them showed consistent expression patterns with the digital gene expression (DGE) data.

**Conclusions:**

A number of genes were differentially expressed between the young floral buds of sterile and fertile plants. Some of these genes may be candidates for future research on CMS in *Nsa* line, fertility restoration and improved agronomic traits in NR1 line. Further study of the unknown tags which were specifically expressed in Fer will help to explore desirable agronomic traits from wild species.

## Background

The novel allo-cytoplasmic male sterility (CMS) system, *Nsa* CMS line, and the corresponding restorer system, NR1 line, have been successfully developed from somatic hybrids between *Brassica napus* (oilseed rape) and its wild relative *Sinapis arvensis* (Yeyou 18, Xinjiang wild mustard from northwestern China) by fusing mesophyll protoplasts [1,2]. Yeyou 18, a Chinese wild population cataloged into *S*. *arvensis* based on genetic analyses [3], possesses valuable agricultural traits such as enhanced resistance to *Sclerotinia sclerotiorum*, *Leptosphaeria maculans* and insects, greater tolerance to low temperatures and drought, low contents of erucic acid and glucosinolates [4], as well as a low incidence of pod shattering [5,6]. The *Nsa* CMS line contains the *S*. *arvensis* cytoplasm, which is essentially different from other rapeseed CMS systems such as *ogu*[7], *nap*[8], *pol*[9], *tour*[10] and *hau*[11], based on their origins and molecular characterization [12]. And the cytoplasmic sterile gene in the *Nsa* CMS line was likely derived from the *S*. *arvensis* parent [13]. Furthermore, the *Nsa* CMS line is more stable to temperature changes compared to *pol* and *nap*[2]. NR1, as a *B*. *napusS*. *arvensis* disomic alien addition line, carries one pair of homologous chromosomes from *S*. *arvensis* and 19 chromosome pairs from *B*. *napus*, and displays important agricultural characters which arise from the alien chromosomes, such as fertility restoration ability to *Nsa* CMS line, low erucic acid and low glucosinolate contents, *S*. *sclerotiorum* resistance and pod shattering resistance [14].

Fertile and sterile plants were derived from the self-pollinated offspring of the F_1_ hybrid between the novel restorer line NR1 and the *Nsa* CMS line. Because NR1 contains one *S*. *arvensis* homologous chromosome pair, on which the restorer genes reside, F_1_ hybrids from NR1 crossed to *Nsa* CMS line are monosomic [14]. The fertility segregation was observed in self-pollinated plants of F_1_ hybrid because of the loss of added chromosome, producing fertile and sterile plants, which possess the identical cytoplasmic genetic background arising from *Nsa* CMS line and similar nuclear genetic background arising from *B*. *napus*, except one or two members of the added *S*. *arvensis* alien chromosome pair in fertile plants. Floral morphology of fertile plants are normal, whereas sterile plants have stamens reduced in size, abnormal anthers and no pollen produced.

To elucidate gene expression and regulation caused by the A and C subgenomes, alien chromosome and cytoplasm from *S*. *arvensis* during the development of young floral bud, especially stamens, we performed a genome-wide high-throughput transcriptomic sequencing for young floral buds of sterile and fertile plants. The transcriptome is the complete set and quantity of transcripts in a cell at a specific developmental stage or under a physiological condition, providing information on gene expression, gene regulation, and amino acid content of proteins [15]. Therefore, transcriptome analysis is essential to interpret the functional elements of the genome and reveals the molecular constituents of cells and tissues. Because of the deep coverage and single base-pair resolution provided by the next-generation sequencing instrument, digital gene-expression (DGE), driven by Solexa/Illumina technology, is an efficient method to analyze transcriptome data. Base on genome-wide expression profiles by sequencing, DGE is able to identify, quantify, and annotate expressed genes on the whole genome level without prior sequence knowledge, opening doors to higher confidence target discovery and pathway studies. This technique has also been widely used in plant research. DGE analysis using Solexa sequencing was performed to identify candidate genes encoding enzymes responsible for the *Siraitia grosvenorii* triterpene biosynthesis [16]. High-throughput tag-sequencing analysis based on the Solexa Genome Analyzer platform was applied to analyze the gene expression profiling of cucumber plant and revealed the comprehensive mechanisms of waterlogging-responsive transcription [17]. Using the Solexa sequencing system, the transcriptomes were compared between seedlings of two soybean varieties to find genes associated with nitrogen use efficiency [18]. Early developing cotton fiber was analyzed by deep-sequencing, and differential expression patterns of genes in a fuzzless/lintless mutant were revealed [19]. DGE signatures were also used to study maize development, and the results from that study provided a basis for the analysis of short-read expression data and resolved specific expression signatures that will help define mechanisms of action of the maize RA3 gene [20]. In addition, Solexa / Illumina technology was used to analyze gene expression during female flower development [21] and gene expression of *Sinapis alba* leaves under drought stress and rewatering growth conditions [22]. Overall, the DGE approach has provided more valuable tools for qualitative and quantitative gene expression analysis than the previous micro array-based assays.

Currently, no progress has been made in identifying the genes that specify CMS and fertility restoration in *Nsa* CMS and NR1 system, and little is understood about how expression of these genes influences the physiological, developmental or biochemical processes such that pollen formation is disrupted. In this study, the transcriptomes of young floral buds were compared between fertile and sterile plants using the Solexa sequencing system. By investigating the expression of genes related to young floral bud development, a number of candidate genes that are important in this process were identified. Here, to our knowledge, this study is the first to characterize genome-wide comparative analysis of gene expression in young floral buds between fertile and sterile plants. And these sequencing datasets will serve as a valuable resource for novel gene discovery from Yeyou 18, the Chinese wild population of *S*. *arvensis*, laying the foundation for elucidating the mechanisms of cytoplasmic male sterility, fertility restoration and improved resistance.

## Results

### Selection of fertile and sterile plants

**Figure 1:**
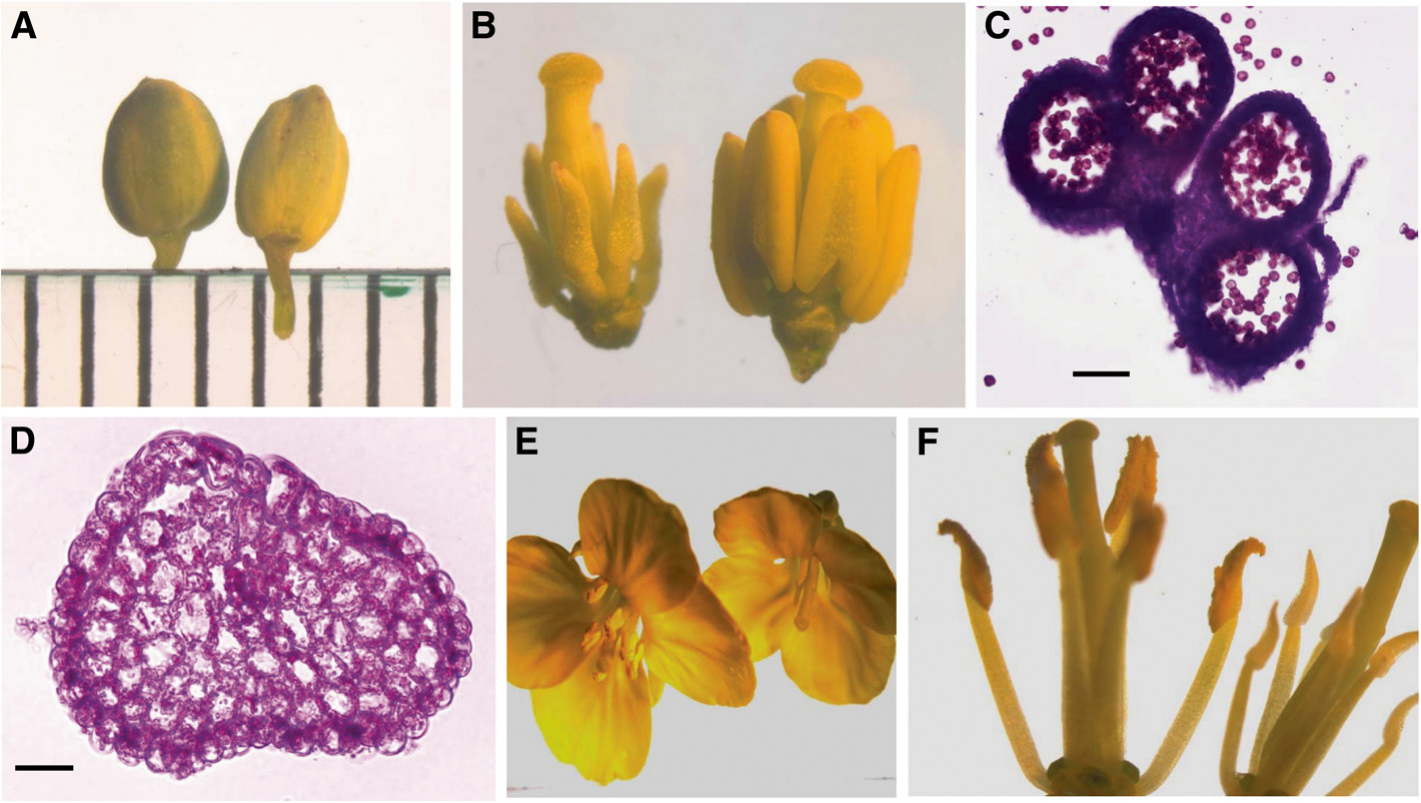
**Comparation of floral morphology between sterile and fertile plants.A**, young buds of sterile and fertile plants (left, fertile; right, sterile); **B**, Anthers corresponding to young buds (left, sterile; right, fertile); **C**, the cross section of fertile anthers; **D**, the cross section of sterile anthers; **E**, mature flowers of sterile and fertile plants (left, fertile; right, sterile); **F**, Anthers corresponding to mature flowers (left, fertile; right, sterile). Scale bars = 50 μm.

To obtain the plant materials with the closest genetic background, fertile and sterile plants were made from the self-pollinated offspring of the F_1_ hybrid between novel restorer line NR1 and *Nsa* CMS line. The cytoplasmic genetic background of fertile plants is the same as that of sterile plants, arising from *Nsa* CMS line. Both the fertile and sterile plants had the complete set of chromosomes from *B*. *napus*, however, there was one or two members of the added *S*. *arvensis* alien chromosome pair in the fertile plants. And thus there were prominently morphological differences appearing at the early anther stage between fertile and sterile buds which were less than 2 mm in diameter (Figure 1A, B). Anthers of fertile bud were larger and fuller than those of sterile bud (Figure 1B). The corresponding cross sections of anthers at this stage showed that in contrast to this early stage in fertile bud, during which four laterally symmetrical locules developed, with two identical adaxial locules and two identical abaxial locules, in sterile bud the loss of locules per anther was evident (Figure 1C, D). At the late anther stage during which mature pollens were released, floral morphology and architecture of fertile plants were normal, whereas sterile plants had defective male flower organs (stamens reduced in size, abnormal anthers and no pollen produced), and had otherwise normal flowers (Figure 1E, F).

CMS-related abnormalities may involve different stages during the formation of male organs and pollen. In many CMS types, the anther development or pollen maturation are impaired. The defect can appear before, during or after meiosis of pollen mother cells resulting in gametophytic or sporophytic CMS types [23]. Although the morphology of normal anther and flower development had been characterized in this study, no detailed morphological comparison of the development of fertile versus sterile anthers was available. To address this, serial sections of fertile and CMS floral buds, representing different development stages of anther, would be compared in further work.

### Solexa sequencing

Table 1 showed that the result of Solexa sequencing and the distribution of distinct clean tags. A total of 4,415,866 and 4,244,140 raw tags were sequenced in sterile plants (Ste) and fertile plants (Fer) libraries, respectively. After filtering out low quality data (tags containing unknown base N and only adaptor tags), 4,199,934 and 4,103,291 tags (designated herein as “clean” tags) remained in Ste and Fer libraries, respectively. To increase the robustness of the approach, single-copy tags in the two libraries (1,439,360 in Ste and 1,388,850 in Fer library) were excluded from further analysis. As a result, a total of 2,760,574 and 2,714,441 clean tags remained from the two libraries, from which 242,163 (Ste) and 253,507 (Fer) distinct tags were obtained. The Fer library had higher number of distinct tags, and showed higher ratio of number of distinct clean tags to total clean tags, and lower percentage of distinct high copy number tags than Ste library. These data suggested that more genes were detected in the Fer library than in the Ste library, and more transcripts were expressed at lower levels in Fer library. There were 11,344 more distinct tags in the Fer than in the Ste library, possibly representing genes related to floral stamen development. The percentage of distinct tags rapidly declined as copy number increased, indicating only a small portion of the transcripts were expressed at high level in the conditions tested. The frequency of these distinct tags was shown in Table 1, which listed the copy numbers in the range 2–100 or higher, in which the majority of distinct tags (more than 80% from each) were present at low copy numbers (< 10 copies), and less than 20% tags from each library were counted between 11 and 100 times. Only approximately 1.3% tags were detected more than 100 times. The data used in this publication have been deposited in NCBI's Gene Expression Omnibus [24] and are accessible through GEO Series accession number GSE42513 (http://www.ncbi.nlm.nih.gov/geo/query/acc.cgi?acc=GSE42513).

**Table 1:** Solexa sequencing tags and the distribution of distinct clean tags in Ste and Fer libraries

	**Ste**	**Fer**
total raw data	4415866	4244140
tags containing N	17065	17032
adaptors	198867	123817
tag copy number =1	1439360	1388850
total clean tag	2760574	2714441
total distinct tag	242163	253507
2–5	162169	170723
6–10	32958	35528
11–20	22111	23436
21–50	16065	15826
51–100	5383	4848
>100	3477	3146

### Annotation analysis of the distinct tag

The full genome sequence for *B*. *napus* is not available. As described by ‘U’s triangle’, the three diploid species *B*. *rapa* (AA genome), *Brassica nigra* (BB genome) and *B*. *oleracea* (CC genome) formed the amphidiploid species *B*. *juncea* (AABB genome), *B*. *napus* (AACC genome) and *B*. *carinata* (BBCC genome) by hybridization [25]. In addition, there was a close relationship between the *S*. *arvensis* and *B*. *nigra*[26]. Therefore, to identify the genes corresponding to the distinct tags in each library, all distinct sequencing tags were annotated using all possible CATG+17-nt sequences of the genome and transcriptome of *B*. *rapa*[27] and those of *B*. *oleracea* (data not shown, the *B*. *oleracea* Genome Project headed by the Oil Crops Research Institute of the Chinese Academy of Agricultural Sciences) as the reference sequences, respectively, allowing only a 1-bp mismatch (Table 2). Altogether, 45, 557 genes (99.56%) and 40, 908 ones (99.35%) had the CATG sites, resulting in a total number of 291, 392 and 282, 216 unambiguous reference tags, respectively, for *B*. *oleracea* and *B*. *rapa*. By assigning the experimental distinct Solexa tags to the virtual reference ones, we observed that 67, 633 (27.93%) and 72, 088 (28.44%) tags were perfectly matched to the *B*. *oleracea* reference genes, 60, 153 (24.84%) and 64, 735 (25.54%) tags were perfectly matched to the *B*. *rapa* reference genes, respectively, in Ste and Fer libraries. Out of the tags matched to reference genes, approximately 10% were mapped to multiple locations, including low complexity tags with poly(A) tails and tags which derived from repetitive sequences and were mapped to highly conserved domains shared by different genes. In addition, approximately 3% tags in two libraries were mapped to the antisense strands, demonstrating that those regions might be bidirectionally transcribed. The tags unmatched to the reference genes were then blasted against the genome of *B*. *rapa* and *B*. *oleracea*, respectively, and approximately 10% tags were matched to the genomic sequences in the two libraries. These might represent non-annotated genes or noncoding transcripts that derived from intergenic regions. As a result of the significant sequencing depth of Solexa technology, incomplete annotation of the genome of *B*. *rapa* or *B*. *oleracea* and one added *S*. *arvenis* chromosome, however, about 25% unmatched tags in each library were observed.

**Table 2:** **Annotation of distinct Solexa tags against reference sequences of*B. rapa *and***B. oleracea, ***respectively**

	**Ste**				**Fer**			
**Reference sequences**	***B.oleracea***		***B.rapa***		***B.oleracea***		***B.rapa***	
Clean distinct tag	242163		242163		253507		253507	
Perfect match(Sense)								
1 tag->1 gene	67633	27.93%	60153	24.84%	72088	28.44%	64735	25.54%
1 tag->n gene	9238	3.81%	7449	3.08%	9908	3.91%	7975	3.15%
1 bp Mismatch(Sense)								
1 tag->1 gene	58766	24.27%	55215	22.80%	58677	23.15%	55444	21.87%
1 tag->n gene	18495	7.64%	16709	6.90%	17955	7.08%	16434	6.48%
Perfect match(Antisense)								
1 tag->1 gene	4560	1.88%	4714	1.95%	4400	1.74%	4581	1.81%
1 tag->n gene	935	0.39%	809	0.33%	839	0.33%	728	0.29%
1 bp Mismatch(Antisense)								
1 tag->1 gene	1770	0.73%	2395	0.99%	1809	0.71%	2530	1.00%
1 tag->n gene	342	0.14%	323	0.13%	368	0.15%	290	0.11%
Mapping to genome								
Perfect match								
1 tag->1 position	9375	3.87%	10515	4.34%	9904	3.91%	11025	4.35%
1 tag->n position	933	0.39%	512	0.21%	1045	0.41%	582	0.23%
1 bp Mismatch								
1 tag->1 position	10448	4.31%	13951	5.76%	11326	4.47%	14545	5.74%
1 tag->n position	2140	0.88%	1560	0.64%	2327	0.92%	1817	0.72%
Reference genes	45758		41174		45758		41174	
Unambiguously matched genes	23909	52.25%	23206	56.36%	25495	55.72%	24819	60.28%
Unknown distinct tag	57528	23.76%	67858	28.02%	62861	24.80%	72821	28.73%

To estimate whether or not the sequencing depth was sufficient for the transcriptome coverage, the sequencing saturation was analyzed in two libraries. The genes that were mapped by all clean tags and unambiguous clean tags increased with the total number of tags. However, when the sequencing counts reached 2 million tags or higher, the number of detected genes was saturated (Additional file 1: Figure S1).

### Differential gene expression between the Ste and Fer libraries

**Figure 2:**
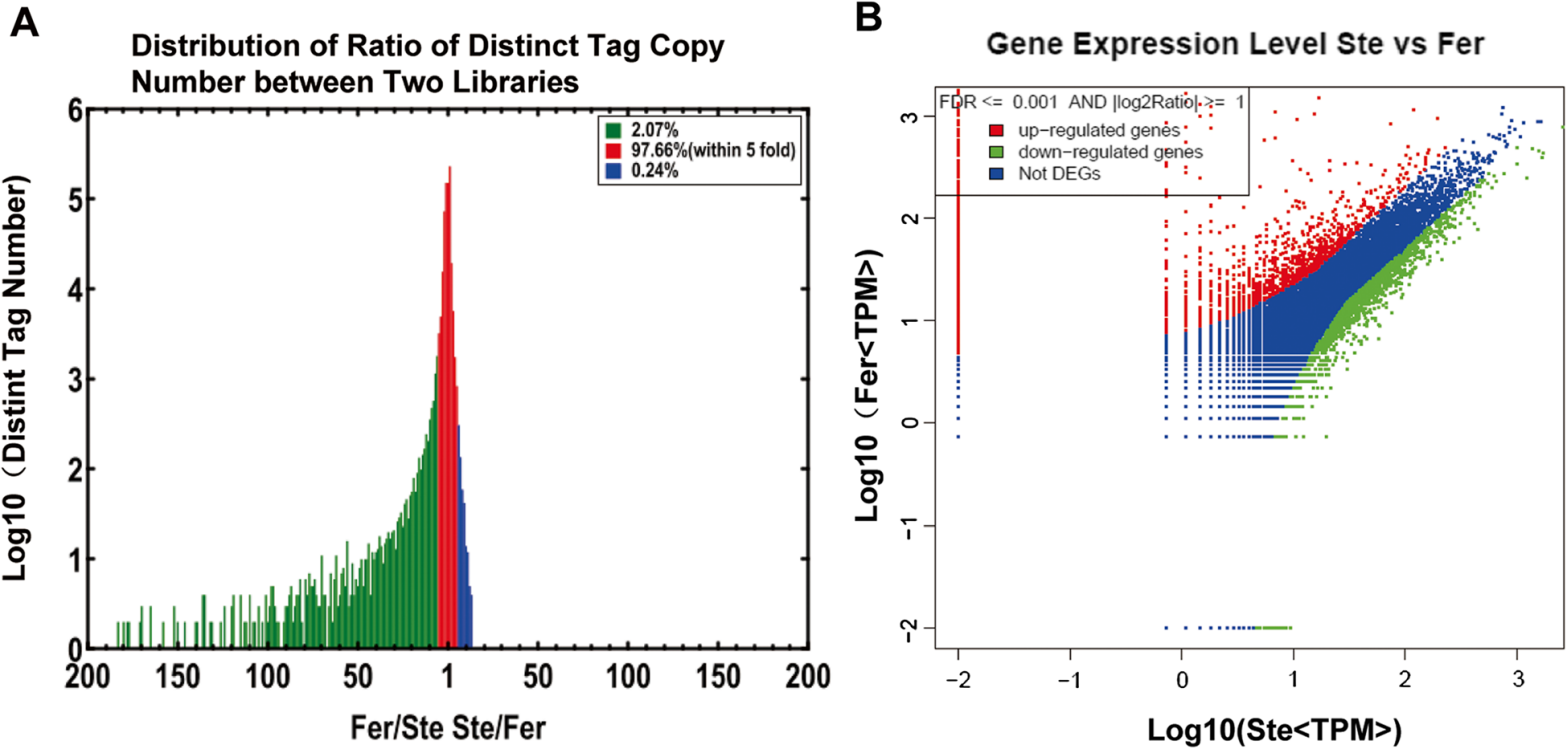
**Differentially expressed tags and corresponding genes in Ste and Fer.A**, Differentially expressed tags in Ste and Fer. The “x” axis represents fold-change of differentially expressed unique tags in two libraries. The “y” axis represents the number of unique tags (log10). Differentially accumulating unique tags with a 5-fold difference between libraries are shown in the red region (97.66%). The blue (0.24%) and green (2.07%) regions represent unique tags that are up- and down-regulated for more than 5 folds in the Fer and Ste libraries, respectively. **B**, Comparision of gene expression levels between the two libraries. For comparing gene expression levels between the two libraries, each library was normalized to 1 million tags. Red dots represent transcripts more prevalent in Fer library, green dots show those present at a lower frequency in Fer and blue dots indicate transcripts that did not change significantly. The parameters “FDR <0.001” and “log2 Ratio ≥ 1” were used as the threshold to judge the significance of gene expression difference.

**Figure 3:**
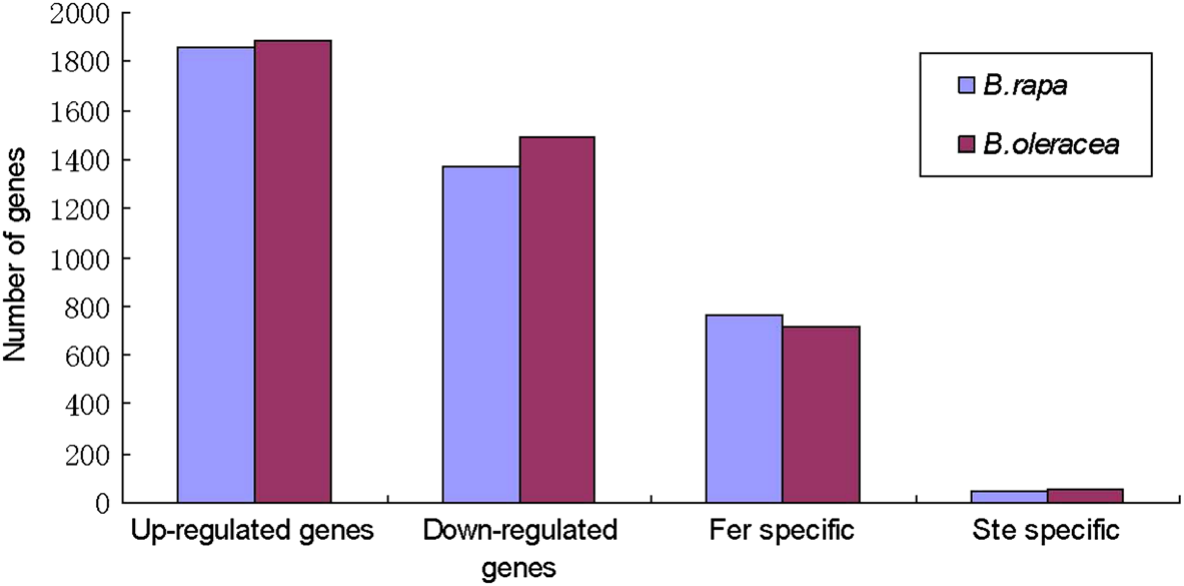
**Changes in gene expression in Fer and Ste libraries.** Numbers of up-regulated, down-regulated, specific to Fer and specific to Ste genes were summarized.

Based on “The significance of digital gene expression profiles” [28], a rigorous algorithm was developed to identify the differentially expressed genes (DEGs) in the two samples. The expression abundance of tag mapped genes in the data sets was analyzed by counting the number of transcripts per million (TPM) clean tags. First, the read density measurement was normalized as described in detail by Benjamini and Yekutieli [29]. FDR ≤ 0.001 and the absolute value of |log2Ratio|≥ 1 were as thresholds to judge the significance of differences in transcript abundance. Differences of tag frequencies that appeared in the Ste and Fer libraries were used for estimating gene expression levels. The distribution of fold-changes in tag number between the two libraries was shown in Figure 2A. The great majority of tags were observed at similar levels, showing a < 5-fold difference in the two libraries. Tags with five folds or greater differences in accumulation accounted for 2.31%, including 2.07% tags which increased by at least five folds and 0.24% tags which were decreased by at least five folds in the Fer library (Figure 2A). The transcripts detected with at least two-fold differences in the two libraries were shown in Figure 2B (FDR <0.001). The red dots and green dots represented transcripts higher or lower in abundance for more than two folds in Fer library, respectively. The blue dots represented transcripts that differed less than two folds between the two libraries, which were arbitrarily designated as “no difference in expression” (Figure 2B). By comparing our two Solexa libraries, a great number of differentially expressed transcripts were identified. And based on above a rigorous algorithm, there were 3231 genes of *B*. *rapa* (Additional file 2) and 3371 genes of *B*. *oleracea* (Additional file 3) which were detected with significant differential expression levels. These included both up-regulated and down-regulated genes in Fer. Of the 1855 up-regulated *B*. *rapa* genes, 760 genes were uniquely expressed in Fer, and 44 of the 1376 down-regulated genes were only expressed in Ste, whereas there were 717 genes of the 1886 up-regulated *B*. *oleracea* genes, which were specially expressed in Fer, and 54 of the 1485 down-regulated genes were only expressed in Ste (Figure 3). In addition, there were 1089 specially expressed tags in Fer, which were neither mapped to *B*. *oleracea* nor mapped to *B*. *rapa*, and these unique tags were presumed to localize on the alien chromosome (Additional file 4).

### Gene Ontology functional analysis of DEGs

**Figure 4:**
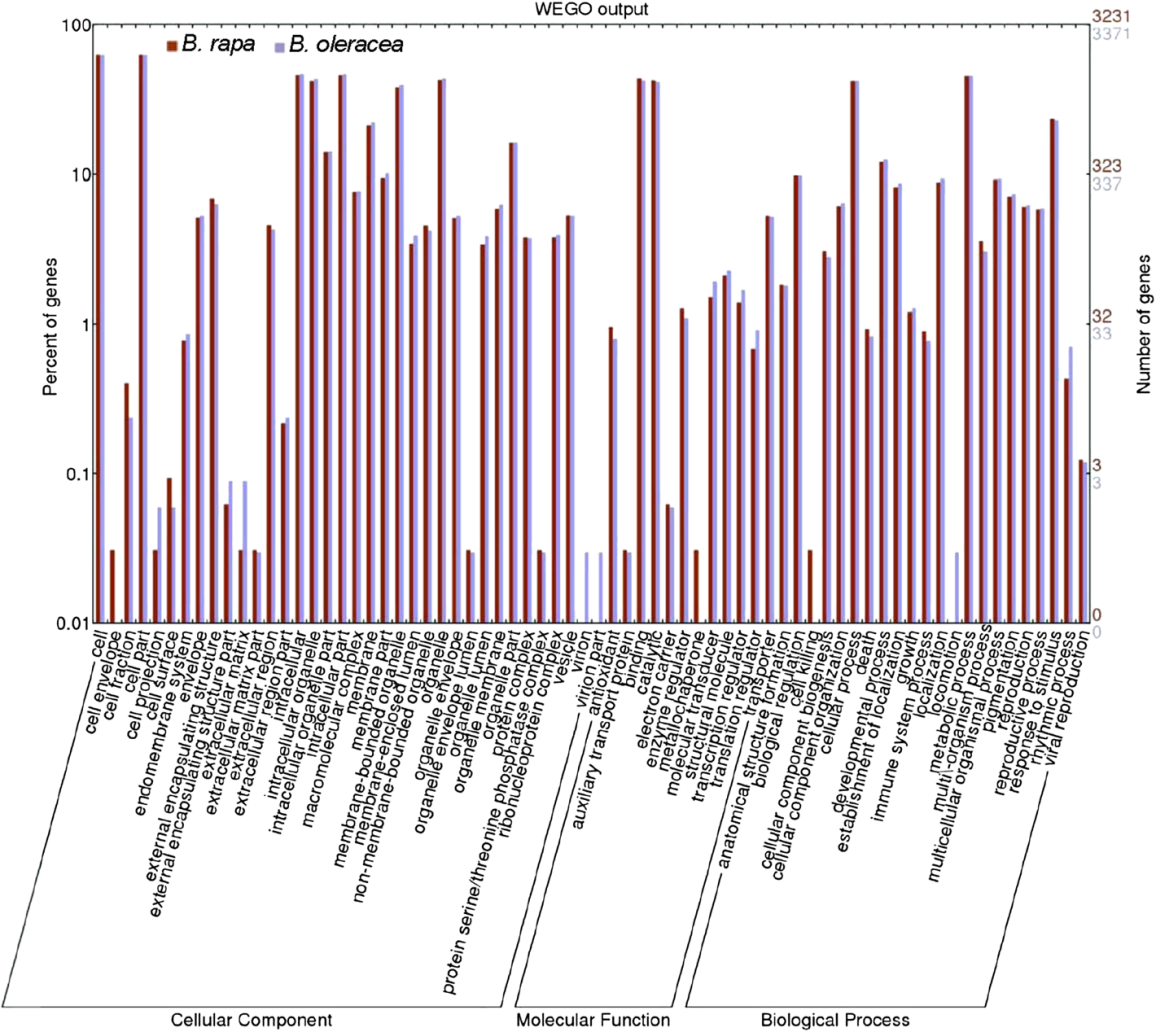
**Histogram showing Gene Ontology functional analysis of DEGs.** The frequency of GO terms was analyzed using GO Slim Assignment. The y-axis and x-axis indicate the names of clusters and the ratio of each cluster, respectively.

Gene Ontology (GO) is an international standardized gene functional classification system that describes properties of genes and their products in any organism, containing three ontologies: molecular function, cellular component and biological process. A total of 3,231 DEGs of *B*. *rapa* and 3371 DEGs of *B*. *oleracea* that could be categorized into 70 categories were found (Figure 4). These DEGs were involved in biochemistry, metabolism, growth, development, and apoptosis. Among the biological process category, metabolic process (about 45%) was the most dominant group, followed by cellular process (about 41%), response to stimulus (about 23%), and developmental process (about 12%). There were two special processes, including cell killing which was unique to *B*. *rapa*, and locomotion which was unique to *B*. *oleracea*. Regarding molecular functions, 43.3% of the *B*. *rapa* unigenes and 42% of the *B*. *oleracea* unigenes were assigned to binding, followed by catalytic activity (42.1% for *B*. *rapa*, 41.3% for *B*. *oleracea*), transporter activity (5.3% for both), and structural molecule activity (2.1% for *B*. *rapa*, 2.3% for *B*. *oleracea*). And metallochaperone function was unique to *B*. *rapa*. In the cellular compo-nent category, cell and cell part (62.4% for both) were the dominant groups, followed by intracellular (45.9% for *B*. *rapa*, 46.7% for *B*. *oleracea*) and intracellular part (45.8% for *B*. *rapa*, 46.5% for *B*. *oleracea*).

To reveal significantly enriched GO terms in DEGs comparing to the genome background, GO enrichment analysis of functional significance was performed. The GO term with P ≤ 0.05 was defined as significantly DEGs enriched GO term. This analysis allowed us to determine the major biological functions, biological processes and cellular components with which DEGs were associated. For molecular function, significantly enriched GO terms of DEGs in *B*. *rapa* (Additional file 5) and *B*. *oleracea* (Additional file 6) included transferase activity (transferring acyl groups), aldehyde-lyase activity and oxidoreductase activity, and zinc ion transmembrane transporter activity was specific to *B*. *rapa*. For enriched biological processes, there were 19 GO terms of DEGs from *B*. *oleracea* (Additional file 7) and 28 GO terms of DEGs from *B*. *rapa* (Additional file 8), which participated in morphogenesis (pollen wall assembly, pollen exine formation, cellular component assembly involved in morphogenesis, anatomical structure formation involved in morphogenesis), development (pollen development, gametophyte development), metabolic process (small molecule metabolism, fatty acid biosynthetic metabolism, alcohol metabolic metabolism), ion transport (cadmium ion transport, di-, tri-valent inorganic cation transport), lipid localization and response to stimulus (response to metal ion, response to inorganic substance).

### Pathway analysis for DEGs

**Figure 5:**
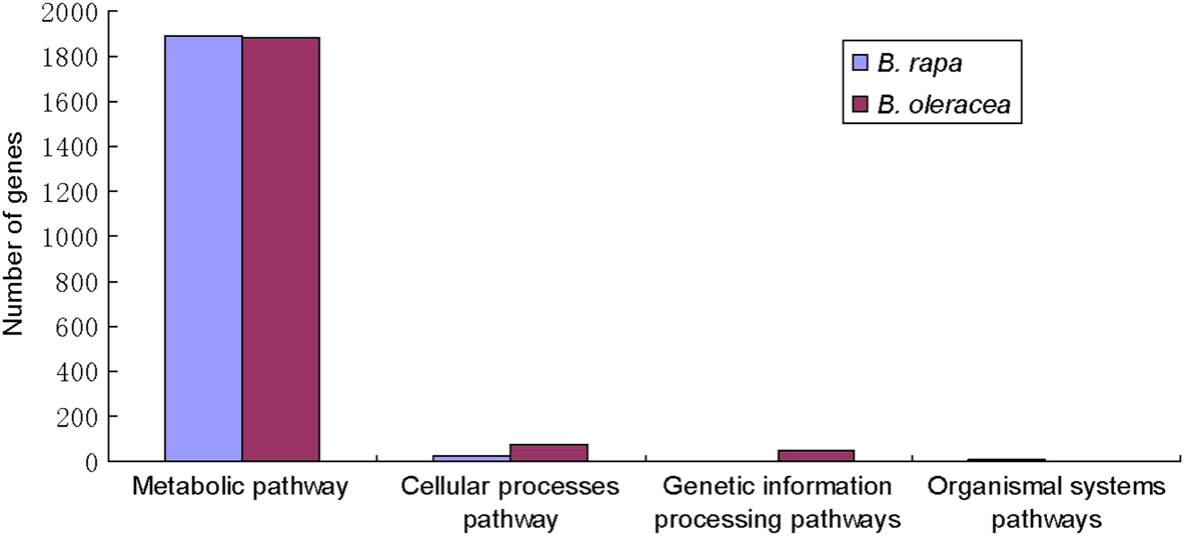
**The summary enriched pathways of DEGs in *B. rapa *and *B. oleracea.***

Different genes cooperate to achieve their biological functions. Pathway-based analysis helps to further understand the biological functions of DEGs. Biological pathways, including metabolic pathways, signal transduction pathways, protein transport and degradation pathways, and genetic information processing pathways, were identified by KEGG pathway analysis of the DEGs, using the major public pathway-related database [30]. Obtained pathways corresponding to DEGs from *B*. *oleracea* and *B*. *rapa* were listed in Additional files 9 and 10, respectively. Significantly enriched pathways that up-regulated and down-regulated genes were involved in were identified by the same statistical algorithms used in the GO analysis. In this study, the enriched pathways were ‘metabolic pathways’ (1888 members for *B*. *rapa*, 1,886 for *B*. *oleracea*) which were large complexes comprising several metabolism patterns, such as ‘amino acid metabolism’, ‘carbohydrate metabolism’, ‘nitrogen metabolism’ and ‘biosynthesis of other secondary metabolites’, ‘cellular process pathways’ (79 members for *B*. *oleracea*, 22 for *B*. *rapa*), ‘genetic information processing pathways’ (51 members for *B*. *oleracea*) and ‘organismal systems pathways’ (9 members for *B*. *rapa*) (Figure 5). And genomic manipulation of these genes which were involved in pathways might be important for elucidate the mechanisms of cytoplasmic male sterility, fertility restoration and improved resistance.

### Expressed genes specific to sterile and fertile plants

**Figure 6:**
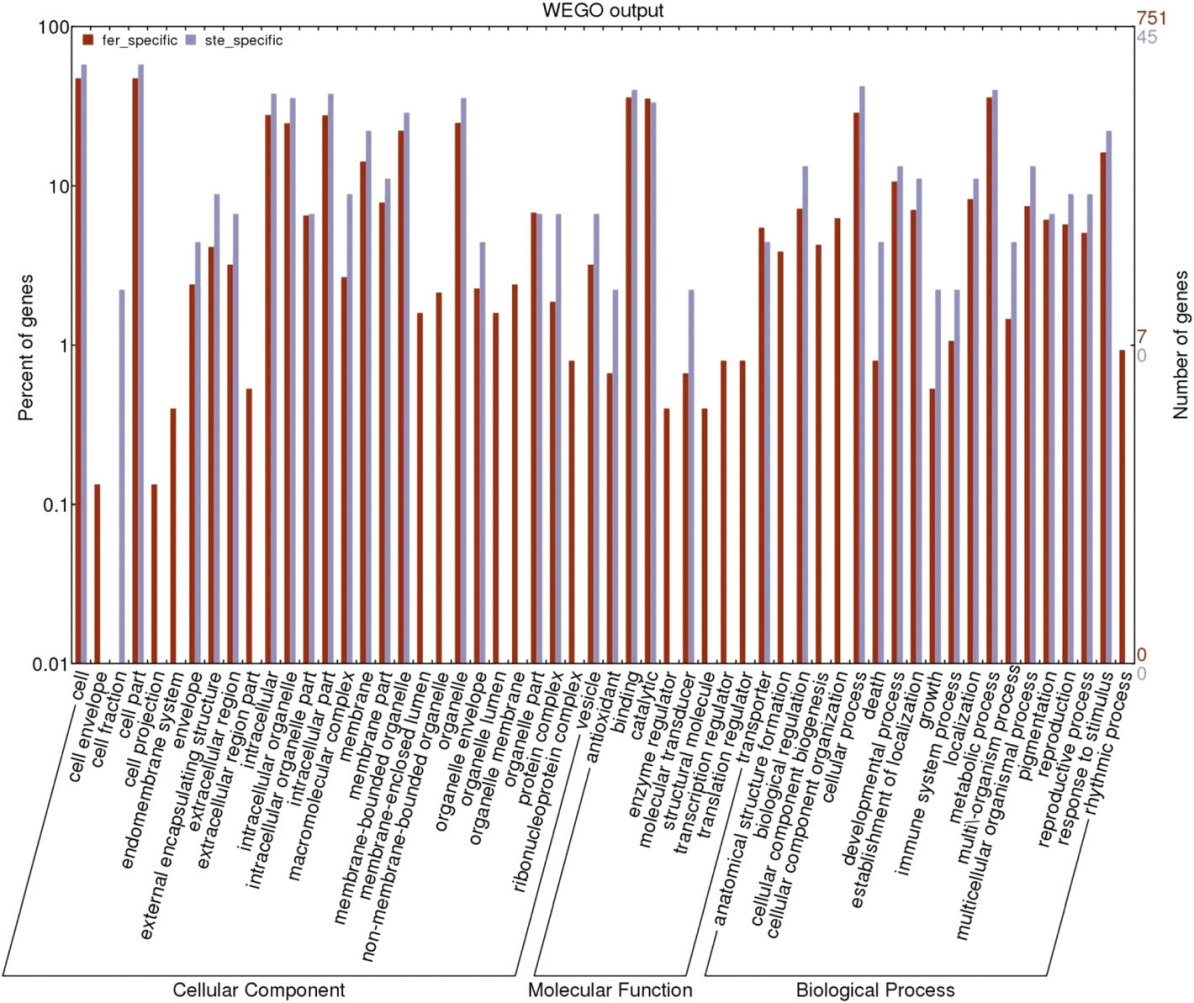
**Histogram showing GO analysis of DEGs which specific to Fer or Ste in *B. rapa.***

Fertile plants carry one or two members of an added homologous chromosome pair from *S*. *arvenis* besides the complete set of chromosomes of *B*. *napus* compared with sterile plants. It was proposed that DEGs specific to fertile plants comprised genes associated with floral development and fertility restoration, whereas DEGs specific to sterile plants may be involved in CMS. Using *B*. *rapa* as reference, a total of 760 DEGs were only expressed in fertile plants, and 44 DEGs were specific to sterile plants, while using *B*. *oleracea* as reference, a total of 717 DEGs were only expressed in fertile plants, and 54 DEGs were specific to sterile plants (Figure 3). By comparing the GO terms of these DEGs, for cellular component, GO terms which were exclusive to fertile plants contained cell envelope, cell projection, endomembrane system, extracellular region part, membrane-enclosed lumen, non-membrane-bounded organelle, organelle lumen and organelle membrane, whereas only cell fraction GO term was specific to sterile plants. For molecular function, GO terms only belonging to fertile plants were enzyme regulator, structural molecule, transcription regulator and translation regulator. For biological process, four GO terms were specific to fertile plants, including anatomical structure formation, cellular component biogenesis, cellular component organization and rhythmic process. No GO terms were specific to sterile plants for molecular function and biological process. The above results were shown in Figure 6.

### DEGs encoding transcription factors

Transcription factors are essential for the regulation of gene expression. Changes in gene transcription are associated with changes in expression of transcription factors. Our DGE results showed that among the significantly DEGs, there were forty-five transcription factors for *B*. *rapa*, including fifteen up-regulated and thirty down-regulated genes (Additional file 2). Among up-regulated *B*. *rapa* transcriptional factors, six ones were specific to Fer plants, including calmodulin-binding protein, mutant TFIIF-alpha, TBP1 (TATA binding protein 1), TBP1 (Telomere-binding protein 1, DNA binding factor (AT-HSFB2B, ATHSFA2) (Additional file 2). For *B*. *oleracea*, there were fifty-seven transcription factors whose expression were dramatically altered, including thirty-nine down-regulated and eighteen up-regulated genes among which six ones were only expressed in Fer. And these transcription factors were ATNAC2, ATHSFA2, transcription factor IID-1 and three unknown proteins (Additional file 2). Further study would be needed to reveal the molecular basis of gene expressional regulation.

### Genes involved in carbon metabolism

Many genes involved in carbon metabolism were differentially expressed between Fer and Ste. Altered expressions of numerous genes involved in pentose phosphate pathway, starch and sucrose metabolism, fructose and mannose metabolism and carbon fixation in photosynthetic organisms were observed. For example, in *B*. *rapa*, twenty genes involved in pentose phosphate pathway showed changed expression. Those genes with increased transcript abundance in Fer encoded glucose-6-phosphate dehydrogenase (Bra028474), three proteins transferring phosphorus-containing groups (Bra025751, Bra025751 and Bra025751), transaldolase-like pro-tein (Bra006197), ribose-phosphate pyrophosphokinase 1/phosphoribosyl diphosphate synthetase 1 (PRSI) (Bra005344), members performing aldehyde-lyase activity (Bra028543, Bra017233, Bra009517, Bra019639 and Bra006960), and the TPMs for these transcripts were up-regulated by 1.0 to 2.1-fold. The eighty-four genes involved in starch and sucrose metabolism were differentially expressed including two genes encoding UDP-glucosyltransferase proteins (Bra009830, Bra015364) and six genes encoding carbohydrate phosphatase proteins (Bra019370, Bra031879, Bra015497, Bra022549, Bra038548, Bra028254). Their expressions were increased by 1.4 to 10.2-fold in Fer. In fructose and mannose metabolism, there were five down-regulated genes, including 6-phosphofructokinase protein family (Bra030394, Bra038519, Bra011089, Bra012940) and phosphoglucomutase-like protein (Bra016184 ), and five up-regulated gene including pfkB-type carbohydrate kinase family protein (Bra014540, Bra007452, Bra029158, Bra028279) and fructose-1,6-bisphosphatase (Bra014841).

### DEGs involved in citrate cycle (TCA cycle) and oxidative phosphorylation

**Figure 7:**
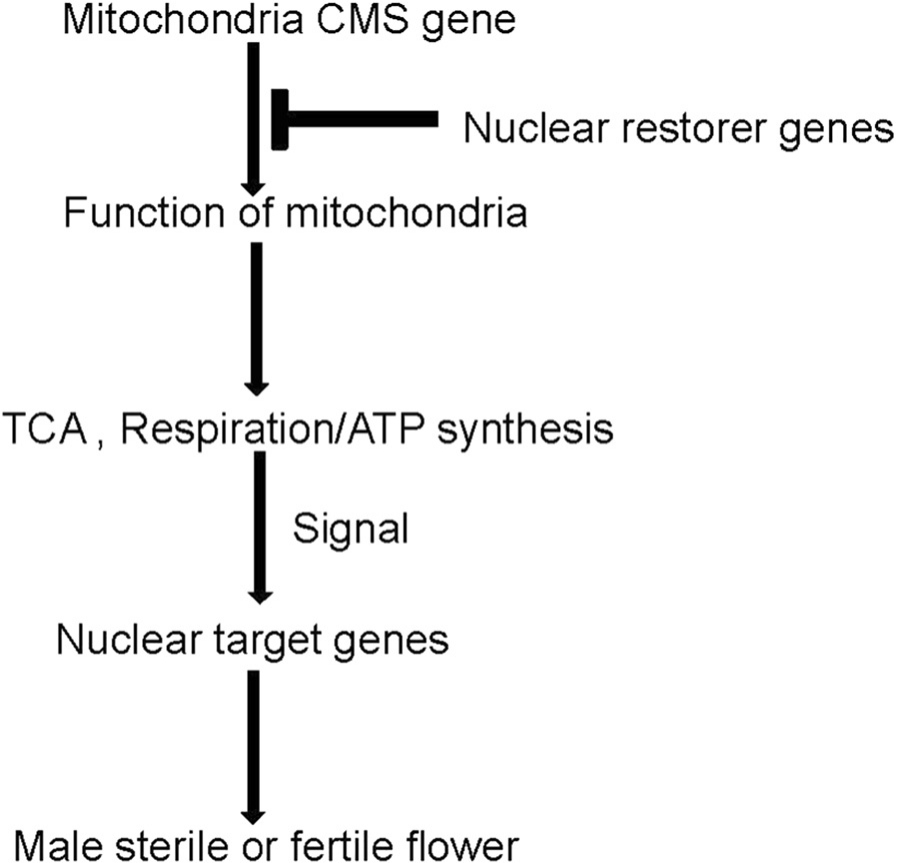
**Overview of CMS in sterile plants and fertility restoration in fertile plants.** Expression of mitochondrial CMS genes led to dysfunction of mitochondria. Products of nuclear restorer genes suppressed the effects of CMS genes. The state of the mitochondria was signaled to nuclear target genes and resulted in male sterile or fertile flower.

Mitochondria are the cellular site of numerous metabolic pathways including the tricarboxylic acid cycle (TCA cycle), respiratory electron transfer and ATP synthesis [31-33]. The amplification, recombination and rearrangement of mitochondrial genomes could cause dysfunction of mitochondria leading to CMS. Altered flower phenotype or defects in pollen formation are presumed to be secondary effects of the mitochondrial mutation, and the primary defect may be a reduction in the efficiency of respiration or the impairment of other mitochondrial function. Using *B*. *rapa* sequences as reference, altered expressions of numerous genes involved in the citrate cycle and oxidative phosphorylation were observed. For example, a total of 12 DEGs were involved in TCA cycle and 11 of which showed decreased transcript abundance in Ste. Down-regulated genes in Ste encoded Class II fumarase (Bra021413), succinate dehydrogenase (Bra033006), PLASTID E2 SUBUNIT OF PYRUVATE DECARBOXYLASE (Bra017345, Bra028057, Bra025167), citrate-synthase (Bra014513, Bra007417), dihydrolipoamide dehydrogenase (Bra001645), IAA-conjugate-resistant 4 (Bra012418), and NADP-specific isocitrate dehydrogenase (Bra004134, Bra038201), whereas malate dehydrogenase 2 (Bra028624) was up-regulated. In addition, twenty DEGs were involved in the oxidative phosphorylation pathway. Nine genes were associated with NADH dehydrogenase, one gene was associated with succinate dehydrogenase/fumarate redutase and ten genes were involved in ATP synthase. Using *B*. *oleracea* sequences as reference, 11 DEGs were involved in TCA cycle and 7 of which performed the same biological function as DEGs from *B*. *rapa* in TCA cycle. According the above results, we then provided an overview about CMS in sterile plants and fertility restoration in fertile plants in Figure 7.

### Genes involved in oxidoreductase activity

In enriched GO terms, oxidoreductase activity was the most enriched term. In *B*. *rapa*, 311 DEGs were involved in oxidoreductase activity, including 26 members acting on the aldehyde or oxo group of donors, NAD or NADP as acceptor. For instance, fatty acyl-CoA reductase (alcohol-forming)/ oxidoreductase (Bra034793, Bra038691), also named MS2 (male sterility 2), acting on the CH-CH group of donors, NAD or NADP as acceptor, was specific to Fer. Others were oxidoreductase, acting on the CH-CH group of donors (Bra006625), cinnamoyl-CoA reductase family (Bra021863, Bra035150), 3-chloroallyl aldehyde dehydrogenase/ aldehyde dehydrogenase (Bra010553, Bra017753, Bra011674, Bra018090, Bra024619 and Bra016330), NAD dependent epimerase/dehydratase family (Bra024073), aldehyde oxidase (Bra002347), NAD or NADH binding/catalytic/ glyceraldehyde-3-phosphate dehydrogenase (Bra026068, Bra026904, Bra040146 and Bra019797), cytosolic factor family protein/phosphoglyceride transfer family protein (Bra026185), cobalamin-independent methionine synthase (Bra023645), and unnamed oxidoreductase members (Bra032939, Bra011470, Bra011869, Bra005012, Bra008743, Bra040146 and Bra023487).

### Differential expressed PPR proteins

Proteins which encode pentatricopeptide repeat (PPR) motif are proposed to function as site-specific, RNA-binding adaptor proteins that mediate interactions between RNA substrates and the enzymes that act on them [34-36]. PPR proteins have a central role in plant mitochondrial biogenesis [37]. Most cloned restorer genes encode mitochondria-targeted PPR proteins [38-44]. In this study, when used *B*. *rapa* as reference sequences, there were nine PPR proteins showed differential expression, including two up-regulated PPR proteins (Bra011721 and Bra017411) and seven down-regulated PPR proteins (Bra033892, Bra008587, Bra025761, Bra036629, Bra031484, Bra033866 and Bra017881) in Fer. These PPR proteins were good candidates for studying the link between nuclear and mitochondrial gene expression.

### Confirmation of tag-mapped genes by qRT-PCR

**Figure 8:**
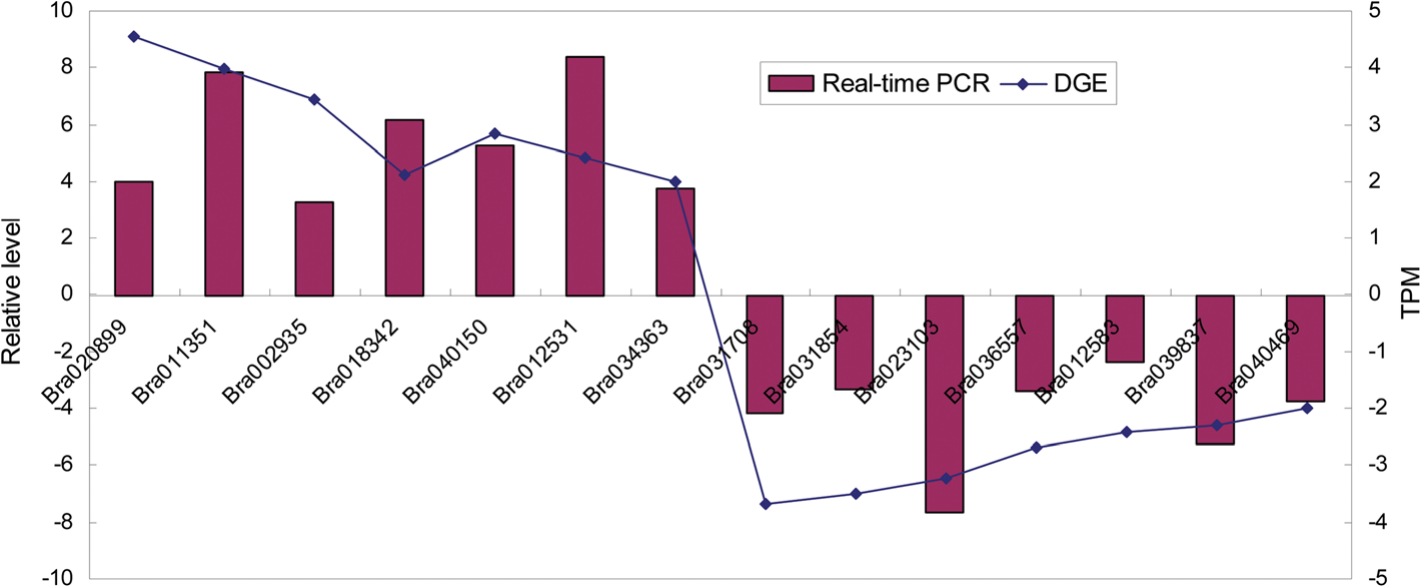
**Real**-**time PCR validations of tag**-**mapped genes.** Relative level, (2−^△CT△CT^); TPM, log2 Ratio (Fer/Ste).

To confirm the reliability of Solexa/Illumina sequencing technology, fifteen genes were randomly selected for quantitative RT-PCR assays. The results showed that expressions of fourteen genes were consistent between the qRT-PCR and the DGE analyses (Figure 8). And the corresponding primers were listed in Table 3. For the gene which showed the inconsistency between qPCR and DGE, it was likely attributable to the fact that DGE was more sensitive in the detection of low-abundant transcripts and small changes in gene expression than qPCR.

**Table 3:** **The corresponding primers of qRT**-**PCR**

**Primer sequences**	**Amplified genes**
ACTAATTTGCACCGTGTT (S)	Bra020899
GGTAGCGTTTCTGTAAGC (A)	
GAAATCTTTGCTTATCCGCTAT (S)	Bra011351
AAACGCTGAGGCACCTTG (A)	
GTCAGATATTTCGGAAGC (S)	Bra002935
CAAGAGTTGAGAACACCC (A)	
GGCGATATTCTCAGTCCA (S)	Bra018342
CACCTCCAAGTGAAACCC (A)	
GCAGGAGGTGCATTAGCC (S)	Bra040150
ACCCAGCAACAACGAAGC (A)	
TGGCTGGGAACAGAACTT(S)	Bra012531
AGGCTTTCCAGACTACGG (A)	
GAAGTCAATGGGAAAGATG (S)	Bra034363
ACTCGAACAGGCTGAAAC (A)	
CTGTCTATTCTCCCTTCCC (S)	Bra031708
AACGGTTTCCCATTGCTG (A)	
AGTCATCGTCGCCGTTAT (S)	Bra031854
AGGATTTGGTCGCAGTCA (A)	
CGACGGATACAGCACAGG (S)	Bra023103
ACGGCTTGGACTCGTTGTAGAT (A)	
AAGCCTCGCCAAAGAAAC (S)	Bra036557
TCAACGGCATGTGAAAGC (A)	
TCTCCGTTACCATCAATA (S)	Bra012583
TGTAGCCATGTCATCTTT (A)	
GAATACGAACGGAGCAAGA (S)	Bra039837
TGGTTTCCCACCATAAGC (A)	
GCTACATACAAGGTCCAGTT (S)	Bra040469
TAAGATAAGCCGCACAAG (A)	
TGCTATCCAGGCTGTTCT (S)	Actin of *B*. *napus*
TTAATGTCACGGACGATTT (A)	

## Discussion

### Global gene transcription changes in Fer and Ste plants

A global analysis of transcriptome could facilitate the identification of systemic gene expression and regulatory mechanisms. In the present study, a transcriptome profiling of young buds was performed to identify genes that are differentially expressed in Fer and Ste plants. A sequencing depth of 4.5 million tags per library was reached (Table 1), and more than 71% unique tags were matched with the genome and transcriptome of *B*. *rapa* and *B*. *olerace*a, respectively, suggesting that the database selected was relatively complete. Because fertile plants carried one chromosome from *S*. *arvensis* besides 19 *B*. *napus* homologous chromosome pairs, exhibiting normal flower morphology and male fertility, more distinct tags were detected in the Fer library than in the Ste library. In addition, for distinct tags, tags with five folds or greater differences in accumulation accounted for 2.31%, however, among which 2.07% tags increased by at least five folds in Fer. This suggested that more genes were involved in gene expression and gene regulation in Fer. Through annotation of distinct tags, there were 1089 specially expressed unknown tags in Fer, which were neither mapped to *B*. *rapa* nor mapped to *B*. *oleracea* (Additional file 4). We presumed that these tags arose basically from the added alien chromosome of *S*. *arvensis*. Further study of these tags would help to dig genes with desirable agronomic traits from wild species. To identify corresponding genes that were associated with the development of stamens (including anthers) and to elucidate the molecular basis of cytoplasmic male sterility, fertility restoration and improved resistance, we analyzed the most differentially regulated tags with a log2 ratio > 1 or < − 1 using a greater statistically significant value (P < 0.001) as well as false discovery rates (FDR < 0.001). And based on the rigorous algorithm, there were 3231 genes of *B*. *rapa* (Additional file 2) and 3371 genes of *B*. *oleracea* (Additional file 3) which were detected with significant differential expression levels. These DEGs were candidates for further research.

### DEGs specific to sterile and fertile plants

Because fertile plants had normal flower morphology and male fertility versus sterile plants, it was proposed that DEGs specific to fertile plants were associated with stamen development and male fertility, whereas DEGs specific to sterile plants may be involved in CMS. Using *B*. *rapa* as reference, a total of 760 DEGs were only expressed in fertile plants, and 44 DEGs were specific to sterile plants. The specific DEGs could be categorized into 56 categories through GO functional analysis, which were involved in biochemistry, metabolism, growth, development, apoptosis and so on (Figure 6). For fertile specific DEGs, 11 DEGs were involved in pollen wall assembly (GO:0010208), including four chalcone synthase genes (Bra000559, Bra034658, Bra011566, Bra017681), three beta-1,3-glucanase genes (Bra028343, Bra037057, Bra038969), two male sterility 2 (MS2) members (Bra034793, Bra038691) and two gycosyl hydrolases family proteins (Bra014979, Bra001918). Chalcone synthase was the key enzyme in the flavonoid biosynthesis pathway, which played an important role in many biological functions in plants, including male fertility [45]. Beta-1,3-glucanase genes were involved in male gametophyte development and pollination. Silencing the expression of beta-1,3-glucanase gene by RNA interference in transgenic rice resulted in male sterility due to disruption of degradation of callose in the anther locules at the early microspore stage [46]. For sterile specific DEGs, 40% of the DEGs were assigned to binding, followed by catalytic activity (33.3%), transporter activity (4.4%), antioxidant activity and molecular transducer activity (2.2% for both) with respect to molecule function category.

### DEGs involved in carbon metabolism and energy metabolism

Mitochondrial gene/genome rearrangements may alter the expression of common mitochondrial genes coding for proteins involved in respiration/ATP synthesis, affecting ATP production and/or other processes within mitochondria. A lowered ATP production [47] or a reduced carbohydrate accumulation [48] was observed in ‘late stage’ CMS flowers. In this study, 11 of 12 DEGs involved in TCA cycle were down-regulated in Ste (Additional file 10). DEGs were also involved in respiration/ATP synthesis and oxidoreductase activity. A total of 20 genes were involved in the oxidative phosphorylation pathway, having effects on NADH dehydrogenase, succinate dehydrogenase/fumarate redutase and ATP synthase (Additional file 10). Oxidoreductase activity was the most enriched GO term (Additional file 5), whereas oxidative stress during microsporogenesis was thought to induce a premature abortion of tapetal cells due to programmed cell death (PCD) in CMS sunflower [49] and rice [50]. In addition, altered expressions of numerous genes involved in pentose phosphate pathway, starch and sucrose metabolism, fructose and mannose metabolism and carbon fixation in photosynthetic organisms were also observed (Additional file 10). The nature of the mitochondrial genes that influence the expression of nuclear genes were unclear. And thus further investigations on these candidate genes help to illuminate the primary targets and downstream components of CMS-associated mitochondrial signaling pathways in *Nsa* CMS line.

### Differentially expressed PPR proteins

Nuclear genomes have crucial roles in mitochondrial biogenesis and function [31,51]. An estimated 10% of eukaryotic nuclear genes encode proteins that are targeted to mitochondria following synthesis on cytosolic ribosomes [32,52]. It has been proposed that the majority of PPR proteins are targeted to plastid or mitochondria [34], and many genetic and biochemical studies conclude that PPRs directly bind to a specific RNA sequence and promote anterograde regulation such as post-transcriptional splicing, processing, editing or regulating mRNA stability [53-60]. From about 450 PPR proteins in *Arabidopsis*, more than 60% are predicted to be targeted to mitochondria [34]. PPR motifs have been suggested to possess binding properties to proteins [61] as well as to RNA [62]. In humans, a PPR related protein with RNA-binding activity is associated with both nuclear and mitochondrial mRNAs and discussed as a potential candidate for a factor that may link nuclear and mitochondrial gene expression [63]. Mutant versions of this protein were shown to be associated with cytochrome c oxidase deficiency in humans [64,65]. Hence, PPR proteins are good candidates for nuclear-encoded factors controlling distinct steps of transcript maturation in mitochondria. Many of them may turn out to be essential players in CMS systems. In addition, PPR proteins have been identified as targets of different miRNAs [66,67]. In this study, when the genome and transcriptome of *B*. *rapa* were used as reference sequences, there were nine PPR proteins showed differential expression, including two up-regulated PPR proteins and seven down-regulated PPR proteins in Fer. These PPR proteins were good candidates for studying the link between nuclear and mitochondrial gene expression.

### Other DEGs

In addition to the genes described above, several other transcript profiles were altered between Ste and Fer. Transcriptional factors play important roles in plant growth and development as well as in hormone and stress responses. A single transcription factor can regulate expression of multiple genes. Therefore, alteration in the expression of transcription factor genes normally results in dramatic changes to a plant [68-70]. As a practical consequence, engineering of transcription factor genes provides a valuable means for manipulation of plants [69]. Our DGE results showed that among the significantly DEGs, there were forty-five transcription factors for *B*. *rapa*, these transcription factors might therefore be potent tools to engineer enhanced stress tolerance in oil crops [71,72].

Two male sterility 2 (MS2) members (Bra034793,Bra038691) were found in Fer, using *B*. *rapa* as referece (Additional file 2). *MS2* gene determines a nuclear male sterile mutant (monogenic recessive) phenotype in *Arabidopsis* and was isolated and characterized using the *En*/Spm-I/dSpm transposon-tagging system [73]. The possible function of the MS2 protein was proposed as a fatty acyl reductase in the formation of pollen wall substances [74]. The homologue of *MS2* in *B*. *napus*, *BnapMS2*, was isolated by cold plague screening from *B*. *napus* anther specific cDNA library [75]. Li et al. [76] isolated a fragment homologous to *BnapMS2* gene from digenic recessive GMS line S45AB of *B*. *napus* using RT-PCR technique, and found that there existed an amino acid divergence between fertile S45B and sterile S45A, which may be the putative male sterility site in S45A. Male sterility gene homolog, designated *BcMS2*, expressed only in stage III flower buds of male fertile Chinese cabbage-pak-choi “ZUBajh97-01B” and there were no detection in all organs of Polima cytoplasmic male sterility (CMS) line ‘Bpol97-05A’ and Ogura CMS line ‘Bogu97-06A’ [77]. In this study, MS2 members (Bra034793, Bra038691) were only expressed in Fer, whose TPM was 1766.48 and 480.76, respectively, and then it was suggested that the two genes were involved in the development of Fer pollens.

Three up-regulated aldehyde dehydrogenase members (Bra024619, Bra018090, Bra016330) were found in Fer, using *B*. *rapa* as referece (Additional file 2). Aldehyde dehydrogenases (ALDHs) have been considered as general detoxifying enzymes which eliminate biogenic and xenobiotic aldehydes in an NAD(P)-dependent manner [78]. Maize nuclear fertility restorer gene *Rf2* as “biochemical restorer,” encoding an aldehyde dehydrogenase, plays an important role in oxidizing both aliphatic and aromatic aldehydes, reducing the amount of a toxic aldehyde and rescuing the developing pollen through a metabolic effect [79,80].

## Conclusions

This study has demonstrated the usefulness of the digital gene expression (DGE) approach to identify the differentially expressed genes (DEGs) between the young floral buds of sterile and fertile plants. In total, there were 3231 genes of *B*. *rapa* and 3371 genes of *B*. *oleracea* which were detected with significant differential expression levels. GO and pathway-based analyses indicate that these DEGs were related to many kinds of molecular functions. In addition, there were 1089 specially expressed unknown tags in Fer, which were neither mapped to *B*. *oleracea* nor mapped to *B*. *rapa*, and these unique tags were presumed to arise basically from the added alien chromosome of *S*. *arvensis*. The results provide a strong basis for future research on CMS in *Nsa* line, fertility restoration and improved agronomic traits in NR1 line. Further work should focus on characterizing these candidate target genes.

## Methods

### Plant materials

The sterile and fertile individual plants were derived from the self-pollinated offspring of the F_1_ hybrid between restorer line NR1 and *Nsa* CMS line, which were grown at the experimental station of the Oil Crops Research Institute, Chinese Academy of Agricultural Sciences, Wuhan, China. For Solexa sequencing, young floral buds (less than 2 mm in diameter) were pooled. A total of 0.5 g materials was snap-frozen in liquid nitrogen and stored at −80°C.

### Frozen section

Stamens were fixed with FAA (10% formalin / 5% acetic acid/45% ethanol/0.01% Triton X-100) for 45 min, and rehydrated through an ethanol series. Stamens were then embedded in OCT compound and frozen, sectioned at 10-μm thickness with frozen section machine Leica CM1850 (Germany). Observations were made with an Olympus microscope and photographed.

### RNA extraction and Solexa/Illumina sequencing

Total RNA from five sterile and fertile individual plants, respectively was extracted using the TRIzol reagent (Invitrogen). After precipitation, RNA was purified with Qiagen’s RNeasy Kit with on-column DNase digestion according to the manufacturer’s instructions. Purified RNA samples were dissolved in diethylpyrocarbonate-treated H_2_O, and the concentration determined spectroscopycally. The quality of the RNA was assessed on 1.0% denaturing agarose gel in combination with the Bioanalyzer 2100 (Agilent). For Solexa sequencing, at least 6 μg of total RNA (≥400 ng/μL) was sent to BGI-Shenzhen, China, following the protocol offered by Illumina for sequencing. The main reagents and supplies were the Illumina Gene Expression Sample Prep Kit and Illumina Sequencing Chip (flow cell), and the main instruments were the Illumina Cluster Station and the Illumina HiSeq™ 2000 System. In details, initially, poly-(A) mRNA was isolated from total RNA sample by poly-(T) oligo-attached magnetic beads. Then, the first- and second-strand cDNAs were synthesized and digested with restriction enzyme *Nla* III, which recognized the CATG sites. Following be washed, the Illumina adaptor 1 was ligated to the 5’ ends of cDNA fragments through the sticky CATG site. Subsequently, cDNA fragments were digested by *Mme* I, as a type of endonuclease with separated recognition sites and digestion sites, which cut at 17 bp downstream of the CATG site, producing tags with adaptor 1. After removing 3'- fragments with magnetic beads precipitation, Illumina adaptor 2 was ligated to the 3'- ends of tags, acquiring tags with different adaptors of both ends to form a tag library. After PCR amplification, purification and denaturation, the single-chain molecules were fixed onto the Illumina Sequencing Chip (Flowcell). Then, four types of nucleotides labeled by four colors were added in, and sequencing was performed via the sequencing by synthesis (SBS) method.

### Processing of sequencing tags and gene expression annotation

Sequencing-received raw image data was transformed by base calling into sequence data, (raw data or raw reads), and was stored in FASTQ format. This type of files stored information about read sequences and quality. Each read was described in four lines in FASTQ files. Raw sequences had 3’- adaptor fragments as well as a few low-quality sequences and several types of impurities. Raw sequences were transformed into clean tags after certain data-processing steps. All clean sequencing tags were annotated using the reference sequences which covered all possible CATG+17-nt tag sequences of the genome and transcriptome of *B*. *rapa* and those of *B*. *oleracea*, respectively using blastn, allowing only a 1-bp mismatch. Clean tags mapped to reference sequences from multiple genes were filtered, and the remaining clean tags were designated as unambiguous clean tags. The number of unambiguous clean tags for each gene was calculated and then normalized to TPM (number of transcripts per million clean tags) [81,82].

### Analysis and screening of DEGs

A rigorous algorithm [28] was used to identify the differentially expressed genes (DEGs) between the two samples. The P-value corresponds to the differential gene expression test. The FDR (False Discovery Rate) is used to determine the threshold of P-value in multiple tests and analyses by manipulating the FDR value. Assume that R differentially expressed genes have been selected, among which S genes truly show differential expression and V genes are false positives. If we decide that the error ratio “Q = V/R” must stay below a cutoff (e.g. 1%), we should preset the FDR to a number no larger than 0.01. FDR ≤ 0.001 and the absolute value of | log2Ratio |≥ 1 were used as thresholds to judge the significance of differences in transcript abundance [29]. More stringent criteria with smaller FDR and greater fold-change value can be used to identify DEGs.

### Real-time quantitative RT-PCR (qRT-PCR) analysis

Real-time quantitative RT-PCR (qRT-PCR) analysis was used to verify the DGE results. The RNA samples used for the qRT-PCR assays were the same as for the DGE experiments. Gene-specific primers were designed according to the reference unigene sequences using the Primer Premier 5.0 (Table S4). qRT-PCR was performed according to the TaKaRa manufacturer specifications (TaKaRa SYBR® PrimeScript™ RT-PCR Kit, Dalian, China). SYBR Green PCR cycling was denatured using a program of 95°C for 10 s, and 40 cycles of 95°C for 5 s and 55°C for 30 s and performed on an iQ™ 5 Multicolor Real-time PCR Detection System (Bio- RAD, USA). *B*. *rapa* actin gene was used as a normalizer, and the relative expression levels of genes were presented by 2^-ΔΔCT^.

### GO and pathway enrichment analysis

GO or pathway enrichment analysis applies a hypergeometric test to identify significantly enriched GO terms or pathways in DEGs in comparison to the whole genome background. The formula used is as follows:

(1) $$P=1-\sum_{i=0}^{m-1}\frac{\left(\begin{array}{c} M\\ i \end{array}\right)\left(\begin{array}{c} N-M\\ n-i \end{array}\right)}{\left(\begin{array}{c} N\\ n \end{array}\right)}$$

For GO enrichment analysis, N is the number of all genes with GO annotation; n is the number of DEGs in N; M is the number of all genes that are annotated to the certain GO terms; and m is the number of DEGs in M. The p value is corrected by Bonferroni, and we chose a corrected-p value ≤ 0.05 as the threshold value. The GO term (P ≤ 0.05) is defined as significantly DEGs enriched GO term. For pathway enrichment analysis, N is the number of genes with a KEGG annotation, n is the number of DEGs in N, M is the number of genes annotated to specific pathways, and m is the number of DEGs in M. The pathways with a Q value of ≤ 0.05 are defined as those with significantly differentially expressed (enriched) genes.

## Competing interests

The authors declare that they have no competing interests.

## Authors’ contributions

XHY contributed to the experimental design and management, data analysis, and manuscript preparation. CHD and JYY contributed to tissue collection, RNA extraction and quantitative RT-PCR, data analysis, manuscript organization and revision. WHL, CHJ, JL and XPF prepared the plant materials. QH provided the NR1 and *Nsa* CMS materials. WHW designed and managed the experiments, organized and reviewed the manuscript. All authors have read and approved the final manuscript.

## Supplementary Material

Additional file 1 **Figure S1.** Sequencing saturation analysis of the two libraries of Ste and Fer. The number of detected genes was enhanced as the sequencing amount (total tag number) increased. A, Ste tags mapped to *B*. *oleracea*; B, Fer tags mapped to *B*. *oleracea*; C, Ste tags mapped to *B*. *rapa*; D, Fer tags mapped to *B*. *rapa.* Click here for file

Additional file 2 **DGEs of *B. rapa.*** Click here for file

Additional file 3 **DGEs of *B. oleracea.*** Click here for file

Additional file 4 **Specially expressed Tags in Fer, which were neither mapped to *B. oleracea *nor mapped to *B. rapa.*** Click here for file

Additional file 5 **Significantly enriched GO terms of DEGs in *B. rapa, *associated with molecular function.** Click here for file

Additional file 6 **Significantly enriched GO terms of DEGs in *B. oleracea, *associated with molecular function.** Click here for file

Additional file 7 **Significantly enriched GO terms of DEGs in *B. oleracea, *associated with biological processes.** Click here for file

Additional file 8 **Significantly enriched GO terms of DEGs in *B. rapa, *associated with biological processes.** Click here for file

Additional file 9 **Pathways corresponding to DEGs from *B. oleracea.*** Click here for file

Additional file 10 **Pathways corresponding to DEGs from *B. rapa.*** Click here for file
