# Supplementary figures and images for: Transcriptome profile analysis of young floral buds of fertile and sterile plants from the self-pollinated offspring of the hybrid between novel restorer line NR1 and Nsa CMS line in Brassica napus
Source: BMC Genomics. 2013 Jan 16;14:26. doi: 10.1186/1471-2164-14-26 (PMC3556089; doi:10.1186/1471-2164-14-26)

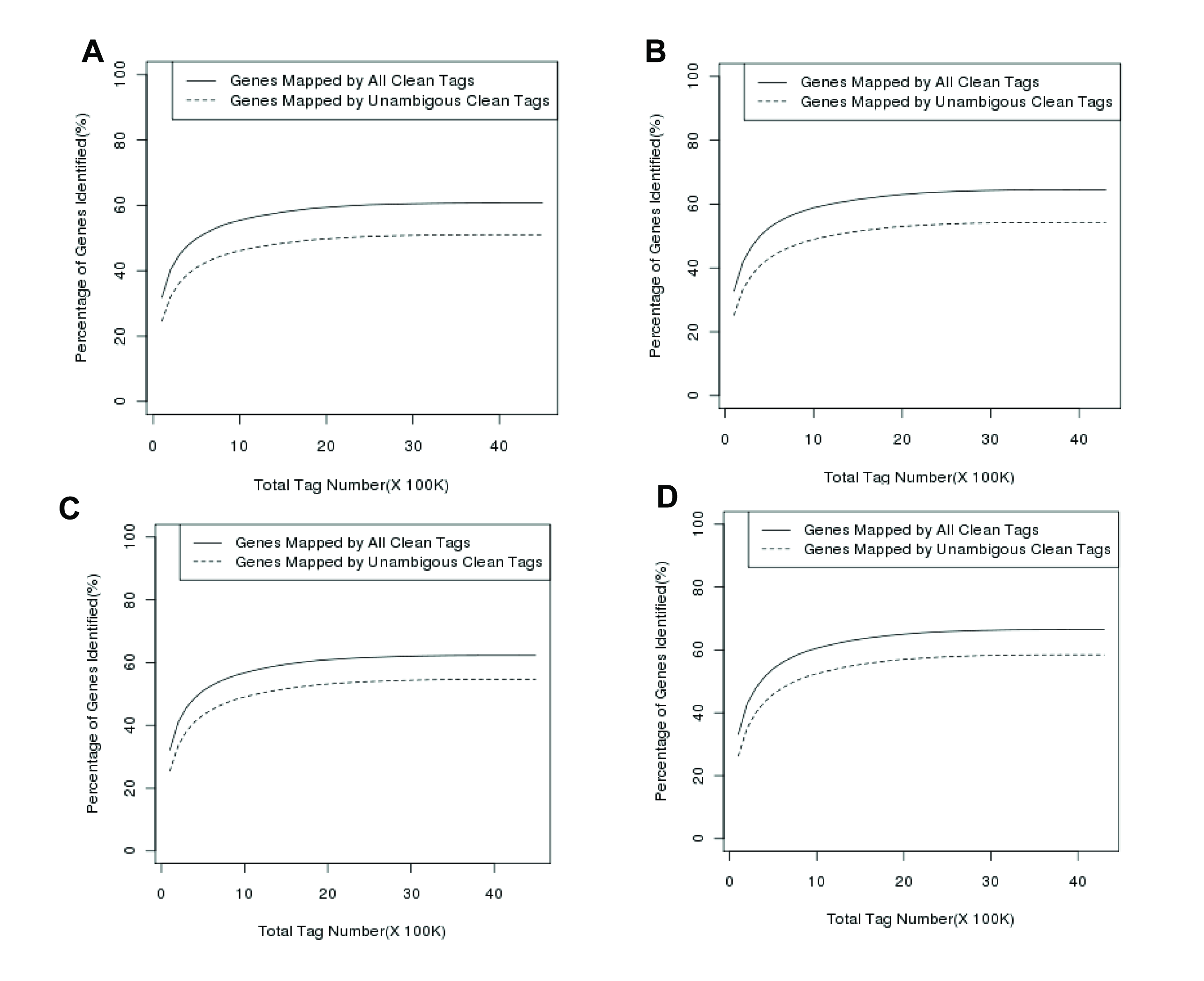

Supplement: Additional file 1 — Figure S1. Sequencing saturation analysis of the two libraries of Ste and Fer. The number of detected genes was enhanced as the sequencing amount (total tag number) increased. A, Ste tags mapped to B. oleracea; B, Fer tags mapped to B. oleracea; C, Ste tags mapped to B. rapa; D, Fer tags mapped to B. rapa. [file 1471-2164-14-26-S1.tiff]
